# Comparative study of epidural ropivacaine and bupivacaine with fentanyl in abdominal surgery

**DOI:** 10.6026/973206300211739

**Published:** 2025-05-31

**Authors:** Asif Kalas, Meghana P Rao, Anwar Hussain

**Affiliations:** 1 Department of Anaesthesiology, KLE JGMMMC, Hubli, KLE Academy of Higher Education and Research, Hubli, Karnataka, India; 2 Department of Anaesthesiology, SDM College of Medical Sciences and Hospital, Dharwad, Karnataka, India

**Keywords:** Epidural analgesia, ropivacaine, bupivacaine, postoperative pain management, abdominal surgery, continuous epidural infusion, hemodynamic stability

## Abstract

The efficacy of equal doses of ropivacaine and bupivacaine with fentanyl as continuous epidural infusions for postoperative analgesia in abdominal surgeries is of interest. Sixty patients were randomized into two groups receiving either ropivacaine or bupivacaine with fentanyl intra-operatively and postoperatively. Sensory and motor blocks, hemodynamic parameters, and pain scores were monitored for 24 hours after surgery. Both groups showed similar sensory and motor block characteristics and recovery profiles with comparable drug dosages. We conclude that ropivacaine and bupivacaine can be used interchangeably as epidural infusions for effective postoperative analgesia with stable hemodynamic and no major complications.

## Background:

Central neuraxial blockade remains the gold standard for anesthesia in lower abdominal surgeries due to its rapid onset and dense, reliable neural blockade. Typically administered into the subarachnoid space between lumbar vertebrae L2-L3 or L3-L4, spinal anesthesia provides effective intraoperative conditions by promoting parasympathetic dominance as the sympathetic block ascends [11, 21]. However, despite its efficacy intra-operatively, postoperative pain management remains a major clinical challenge [3]. Abdominal surgeries are among the most commonly performed procedures and are frequently associated with moderate to severe postoperative pain. Effective pain management requires a multimodal approach, combining systemic analgesics and regional anesthesia techniques, such as thoracic or lumbar epidural analgesia, to minimize the physiological stress response and improve outcomes [4, 5]. Although opioids have historically been central to postoperative pain control, their adverse effects and the rising concern of misuse and dependency have prompt a shift toward opioid-sparing multimodal strategies [6, 7]. Pain is inherently subjective, and individual factors-such as comorbidities and psychosocial influences-further complicate its management [8, 9]. Notably, approximately 75% of surgical patients experience acute postoperative pain, with less than half reporting adequate relief [9, 10]. Inadequate pain control may lead to a range of complications, including delayed recovery, prolonged hospitalization, and even persistent postoperative pain, which affects 2-10% of adults [11]. Among regional techniques, epidural analgesia is recognized as one of the most effective and safest methods, often resulting in shorter ICU stays and superior dynamic pain control. Combining local anesthetics with lipophilic opioids such as fentanyl enhances analgesia, reduces local anaesthetic requirements, and minimizes sensory block regression, without significantly increasing the risk of delayed respiratory depression [12, 13-14]. Studies have shown that lipophilic opioids provide rapid onset and clearance, making them ideal for postoperative epidural administration [15]. Additionally, the benefits of such combinations have been validated in various clinical settings, including abdominal surgery [16]. Therefore, it is of interest to report the comparative study of epidural ropivacaine and bupivacaine with fentanyl in abdominal surgery.

## Materials and Methodology:

We had conducted a prospective observational study at department of anesthesiology, in tertiary care hospital, by including all the patients undergoing abdominal surgeries.

## Inclusion criteria:

[1] We had included all the patients aged between 18 to 70 years of either gender.

[2] Patients belonging to American Society of Anesthesiologists (ASA) I and II.

[3] Patient weighing between 40-90kg.

## Exclusion criteria:

[1] Patients with priorh/neurologic, cardiopulmonary, liver or renal impairments and psychiatric disease.

[2] Pregnant and lactating women, Patients with coagulopathies and increased intracranial tension Patient allergic to drugs used in study having contra indications to epidural anesthesia and abdominal trauma.

## Study population:

Cases undergoing abdominal surgeries and full filling inclusion criteria during study period will be randomly allocated in any group. All patients had undergone pre-anesthesia evaluation. Patients who fulfilled inclusion criteria were enrolled for this study. No analgesic was given on the day of surgery. Familiarize with the recording of post-operative pain using a 10-cm visual analogue sale anchored at one end by no pain at all and at the other end by worst pain imaginable. After induction group B received 0.25% bupivacaine with 2mcg/cc fentanyl (8ml) as bolus dose via epidural catheter. After one hour, epidural infusion was started with 0.25 % bupivaciane with 2mcg/cc fentanyl at a rate adjusted to hemodynamics parameters whereas group R received ropivaciane. These infusions were stopped half an hour prior to expected time of extubation. Patients were reversed after meeting extubation criteria with neostigmine and glycopyrolate.

## Statistical analysis:

All the obtained results were tabulated in Microsoft Excel and analysed by using suitable statistical test by SPSS 23.0 version. Descriptive data of demographic details were analyzed by mean, standard deviation and percentages. Comparisons of the parameters between two groups were assessed by using student t test: paired. Obtained results have been represented as tables and graph below. Group R represents Ropivacaine group and Group B is the patient group administered with Bupivacaine.

## Results:

There were 18 (60%) males and 12 (40%) male and female in group R. 17 males and 13 females in Group B, with no significant difference in the distribution of gender between both groups (Figure 1 - see PDF). The same is represented as bar diagram below. Comparison of epidural infusion with 0.125% ropivacaine and bupivacaine with fentanyl for postoperative analgesia in abdominal surgeries: A prospective randomized double-blind study (Table 1, Figure 2 - see PDF). From the above table we could observe that the distribution of age also was almost similar in both groups. Majority of them were aged between 31 to 40 years with incidence of 40% followed by those aged between 21 to 30 years (Table 2). Above are the average baseline vital parameters, we could observe that there was no changes statistically significant difference in baseline vital parameters (Table 3). From Mann Whitney Utestit can be observed that, there is no significant difference in mean of any variable of VAS over groups (Table 4). Even there covery of the motor block assessed by using Bromage scale was also not significantly different between two groups (Table 5, Figure 3 - see PDF).

## Discussion:

Epidural analgesia is a time-tested anesthetic technique that provides pre-emptive analgesia, thereby effectively preventing central sensitization, limiting the requirement for multiple systemic drugs (polypharmacy), and facilitating early physiotherapy and ambulation in the postoperative period [17, 18, 19]. This contributes significantly to the principles of enhanced recovery after surgery (ERAS). The ability to provide segmental analgesia while preserving motor function and hemodynamic stability has made epidural analgesia the gold standard for many abdominal surgeries [20, 21]. A widely practiced approach in epidural anesthesia involves the combination of local anesthetics with lipophilic opioid adjuvants, such as fentanyl. Fentanyl, due to its lipophilic nature, exerts rapid analgesic effects and is less likely to migrate cephalad, thus minimizing the risk of respiratory depression-a risk more commonly associated with hydrophilic opioids like morphine [22, 23-24]. Additionally, opioids like fentanyl potentiate the effects of local anesthetics, enabling the use of lower doses, which in turn reduces side effects and systemic toxicity [25]. Traditionally, bupivacaine has been the local anesthetic of choice due to its long duration of action. However, ropivacaine, a newer agent, has gained attention due to its improved safety profile. Structurally similar to bupivacaine, ropivacaine is the S-enantiomer of an amide-type local anesthetic and is characterized by a higher ionization constant and lipid solubility, resulting in selective sensory blockade with minimal motor involvement [261, 27-28]. Its selective action on A-delta and C fibers, which mediate pain transmission, more than on A-beta fibers responsible for motor activity, explains its relative motor-sparing properties at low concentrations [29]. Importantly, ropivacaine has been shown to have a significantly lower cardiotoxicity and higher threshold for central nervous system toxicity than bupivacaine. This makes it a preferred choice in continuous epidural infusion, especially for high-risk surgical patients [30, 31-32]. Our study aimed to assess and compare the efficacy, hemodynamic effects, and adverse events associated with continuous epidural infusion of ropivacaine and bupivacaine, both in combination with fentanyl, in patients undergoing abdominal surgeries. In our study, all participants underwent general anesthesia with epidural catheter placement and received a bolus dose followed by a continuous infusion of the respective drug combinations intraoperatively. Postoperatively, patients were maintained on the same respective drugs via epidural infusion for 24 hours. The demographic parameters, including age and gender, were statistically similar between the two groups, thus ensuring comparability. Additionally, baseline vitals and duration of surgery were not significantly different across groups. This enabled a focused evaluation of drug-specific effects on analgesia, motor block, and hemodynamic stability. When evaluating hemodynamic changes, both systolic and diastolic pressures exhibited mild reductions between the 2nd and 4th postoperative hours. However, this was not statistically significant. Four patients (13.3%) in the bupivacaine group experienced hypotension compared to only one (3.3%) in the ropivacaine group, all managed successfully with ephedrine boluses. These findings are supported by Clarkson who noted that ropivacaine causes less cardiovascular depression due to its reduced impact on myocardial sodium channels [46]. Similarly, Clarksonand Smith *et al.* also reported no significant variation in blood pressure or heart rate in patients receiving either drug during epidural analgesia [46, 48].

Oxygen saturation remained stable throughout in our study cohort. However, in a study by Clarkson *et al.* one patient in the ropivacaine group developed desaturation 16 hours post-surgery, which necessitated intubation and mechanical ventilation [46]. This incident was attributed to possible cephalad migration of the opioid, catheter misplacement, or accumulation of protein-bound drug, underscoring the importance of careful catheter placement, dose regulation, and patient monitoring. Fentanyl, being highly lipophilic, is less likely to produce late respiratory depression compared to morphine [33, 34]. Sensory level achievement to T10 dermatome was more frequent in the bupivacaine group (70%) than in the ropivacaine group (56.6%), likely due to bupivacaine's greater lipid solubility potential for cephalad spread. These results correlate with those observed by Bhat *et al.* who also noted greater cephalad block spread with bupivacaine [11]. The Visual Analog Scale (VAS) scores, used to assess pain intensity, were similar at 0 and 24 hours across both groups. However, from the 4th hour onward, Group R demonstrated clinically lower VAS scores, although the difference was not statistically significant (*e.g.*, 3.23 ± 0.728 vs. 3.37 ± 0.615 at 4 hours, p = 0.502). These results are consistent with those of Fleisher *et al.* who found that ropivacaine provided comparable, if not slightly better, pain relief over bupivacaine when combined with fentanyl [48]. The motor blockade, evaluated by Bromage scoring, revealed that ropivacaine caused earlier regression of motor block. At the 10th postoperative hour, 43.3% of patients in Group R had full lower limb flexion compared to 10% in Group B. By the 12th hour, 70% in Group R and 50% in Group B had regained complete motor function. All patients reached Bromage 0 (no motor block) by 24 hours. These findings again reflect the results of Bhat *et al.* and Patil *et al.* both of whom reported significantly shorter duration of motor block in ropivacaine groups [11, 31]. The underlying reason for this difference lies in the pharmacokinetic and pharmacodynamics profiles of the two agents. Ropivacaine, with lower lipid solubility and selective sensory fiber blockade, achieves adequate analgesia while minimizing motor block [35, 36 and 37]. Additionally, its protein binding characteristics and hepatic metabolism contribute to its predictable and safe profile during continuous infusion [38]. This study reinforces the view that ropivacaine is a safer alternative to bupivacaine in epidural anesthesia, offering equivalent analgesia, better motor recovery, and fewer hemodynamic disturbances. This is consistent with the findings of several earlier studies [39, 40, 41-42, 43, 44, 45, 46- 47]. While more large-scale randomized trials are needed, the current evidence supports ropivacaine's superior clinical profile, especially in multimodal pain management protocols.

## Conclusion:

Ropivacaine 0.125% with fentanyl 2 mcg/kg is as effective as Bupivacaine 0.125% with fentanyl 2 mcg/kg in providing sensory and motor block with comparable hemodynamic stability. Both drugs show similar recovery profiles and 24-hour dosing requirements. Therefore, either drug can be used interchangeably without major complications.

## Figures and Tables

**Table 1:** Distribution of gender of the patients in both groups

**Gender**	**Group R**	**Group B**	**P value**
Male	18 (60%)	17 (56.66%)	0.13
Female	12 (40%)	13 (43.34%)	

**Table 2:** Distribution of age

**Age**	**Group R**	**Group B**
21 to30	9 (30%)	7 (23.3%)
31 to40	12 (40%)	12 (40%)
41 to50	6 (20%)	7 (23.3%)
51 to60	3 (10%)	4 (13.3%)
61 and above	Nil	Nil
Mean ±SD	36.8 ± 9.85	38.36 ± 10.25

**Table 3:** Comparison of baseline vital parameters

**Parameter**	**Group R**	**Group B**	**P value**
Heart rate	98.50±7.8	100.11±3.79	0.14
SBP	126.78±10.82	124.65±8.4	0.2
DBP	68.52±9.8	66.79±10.3	0.11
MAP	101.64±12.5	100.8±8.23	0.31
average duration of the procedure in minutes	112.7 ± 20.68	111.8 ± 19.16	0.43
Average dose required in milligram	21.87±4.9	20.32±3.64	0.58

**Table 4:** Distribution of subjects according to the VAS score

**Time**	**Subcategory**	**Group R**	**Group B**	**p-value**
Baseline	Mean ±SD Median (Min, Max)	8.93 ± 0.907 9 (7,10)	8.93 ± 0.907 9 (7,10)	0.992MW
2 hrs	Mean ±SD Median (Min, Max)	5.87 ± 0.819 6 (4,7)	5.87 ± 0.819 6 (4,7)	0.992MW
4 hrs	Mean ±SD Median (Min, Max)	3.23 ± 0.728 3 (2,5)	3.37 ± 0.615 3 (3,5)	0.502MW
6 hrs	Mean ±SD Median (Min, Max)	1.23 ± 0.858 1.5(0, 2)	1.5 ± 0.938 2 (0,3)	0.214MW
8 hrs	Mean ±SD Median (Min, Max)	0.73 ± 0.828 0.5(0, 2)	0.93 ±0.868 1 (0,2)	0.400MW
12 hrs	Mean ±SD Median (Min, Max)	0.2 ± 0.407 0 (0,1)	0.33 ± 0.479 0 (0,1)	0.373MW
24 hrs	Mean ±SD Median (Min, Max)	0	0	0.992MW

**Table 5:** Distribution of subjects according to BROMAGE grading

**Variable**	**Subcategory**	**Group R**	**Group B**
Baseline	3	30 (100%)	30 (100%)
2 hrs	2	7 (23.3%)	7 (23.3%)
	3	23 (76.7%)	23 (76.7%)
4 hrs	2	30 (100%)	27 (90%)
	3	0	3 (10%)
6 hrs	1	3 (10%)	1 (3.3%)
	2	27 (90%)	29 (96.7%)
8 hrs	1	18 (60%)	14 (46.7%)
	2	12 (40%)	16 (53.3%)
10 hrs	0	13 (43.3%)	6 (20%)
	1	9 (30%)	16 (53.3%)
	2	8 (26.7%)	8 (26.7%)
12 hrs	0	21 (70%)	15 (50%)
	1	7 (23.3%)	13 (43.3%)
	2	2 (6.7%)	2 (6.7%)
24 hrs	0	30 (100%)	30 (100%)
